# Capsules of Cod-liver Oil

**Published:** 1844-04

**Authors:** 


*Capsules of Cod-Liver Oil.*—We have been shown by Mr. Milhau, at his pharmacy, 183 Broadway, beautiful capsules of cod-liver oil, prepared by Mr. W. F. Stone, by means of which the unpleasant taste of this medicine is wholly obviated, Each capsule contains, besides the cod-liver oil, one-twentieth grain of iodine. This oil is a remedy which has recently come into notice, as shown in some of our previous numbers; and, in Germany, it is regarded almost as a panacea.—*Ib.*

